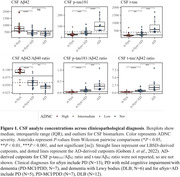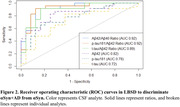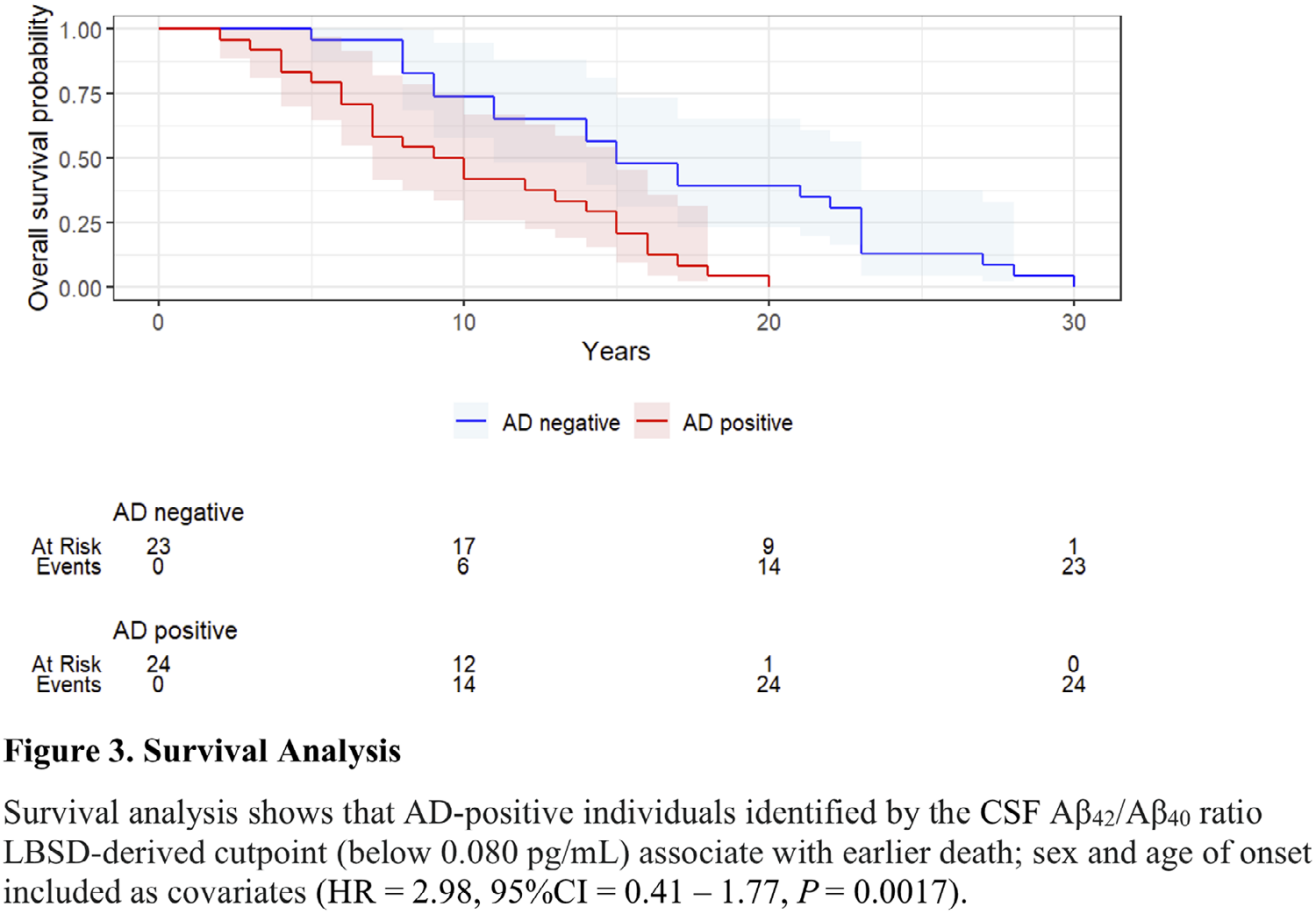# Improving detection of concomitant Alzheimer’s disease pathology in autopsied Lewy body disorders using CSF biomarkers

**DOI:** 10.1002/alz.089913

**Published:** 2025-01-09

**Authors:** Isabela Albuja, David J Irwin, Alice Chen‐Plotkin, Leslie M. Shaw, Eddie B Lee, David A Wolk, Thomas F. Tropea, Katheryn A Q Cousins

**Affiliations:** ^1^ University of Pennsylvania, Philadelphia, PA USA; ^2^ Department of Neurology, University of Pennsylvania, Philadelphia, PA USA; ^3^ Perelman School of Medicine, University of Pennsylvania, Philadelphia, PA USA; ^4^ Dept of Pathology & Laboratory Medicine, University of Pennsylvania, Perelman School of Medicine, Philadelphia, PA USA; ^5^ Department of Pathology & Laboratory Medicine, University of Pennsylvania, Philadelphia, PA USA

## Abstract

**Background:**

While the cerebrospinal fluid (CSF) biomarkers β‐amyloid 1‐42 (Aβ_42_), β‐amyloid 1‐40 (Aβ_40_), total tau (t‐tau), and phosphorylated tau 181 (p‐tau_181_) can detect Alzheimer’s disease (AD) pathology during life, there is a need to evaluate CSF cutpoints for specific application in Lewy body spectrum disorders (LBSD) in order to maximize detection to AD co‐pathology. Using the Fujirebio LUMIPULSE G, we developed LBSD‐derived cutpoints for CSF biomarkers and their ratios (Aβ_42_/Aβ_40_, p‐tau_181_/Aβ_42_, t‐tau/Aβ_42_) to determine which best discriminates αSyn with AD (αSyn+AD) from αSyn without (αSyn). We compared our LBSD‐derived cutpoints to the established AD‐derived cutpoints.

**Method:**

Participants were neuropathology‐confirmed LBSD, with (αSyn+AD; n=24) or without AD co‐pathology (αSyn; n=26). Amnestic AD without αSyn were included as a reference group (n=11). Receiver operating characteristic (ROC) and area under the curve (AUC) tested how accurately biomarkers discriminated between αSyn+AD and αSyn. Optimal cutpoints were identified by Youden's Index using bootstrapping (2000 iterations). Sensitivity, specificity, and accuracy were calculated at best cutpoints. Confidence intervals (95%CI) tested for differences between empirically‐defined LBSD‐specific cutpoints and previously established AD‐derived cutpoints. Chi‐square tests compared classification accuracy between LBSD‐ and AD‐derived cutpoints. Survival analyses tested if biomarker status predicted time to death.

**Result:**

Discriminating between αSyn and αSyn+AD, the CSF Aβ_42_/Aβ_40_ ratio had the highest accuracy (0.90), followed by p‐tau_181_/Aβ_42_ ratio (0.87). We found that LBSD‐derived cutpoints were higher than established AD‐derived cutpoints for CSF Aβ_42_/Aβ_40_ ratio (0.080 [95%CI=0.069 – 0.089] vs. 0.058 pg/mL), and lower for CSF p‐tau_181_ (32.7 [95%CI=26.84 – 38.76] vs. 50.2 pg/mL) and t‐tau (227 [95%CI=203.51‐ 278.66] vs. 409 pg/mL). Furthermore, LBSD‐derived cutpoints identified individuals with AD co‐pathology more accurately than the AD‐derived cutpoints for CSF Aβ_42_/Aβ_40_ (X^2^= 23.26, P<0.001), p‐tau_181_ (X^2^=22.82, P<0.001) and t‐tau (X^2^= 15.09, P<0.001). Survival analysis show that AD‐positive individuals identified by the LBSD‐derived CSF Aβ_42_/Aβ_40_ ratio cutpoint associate with earlier death (HR = 2.98, 95%CI = 0.41 – 1.77, P = 0.0017).

**Conclusion:**

This study helps to establish AD CSF biomarkers for specific application in LBSD using the Fuijirebio LUMIPULSE G platform to maximize detection of AD co‐pathology. Validation in an independent cohort is needed.